# Acute and Preventive Treatment of COVID-19-Related Headache: A Series of 100 Patients

**DOI:** 10.3390/life14070910

**Published:** 2024-07-22

**Authors:** David García-Azorín, Claudia García-Ruiz, Álvaro Sierra-Mencía, Yésica González-Osorio, Andrea Recio-García, Ana González-Celestino, Cristina García-Iglesias, Álvaro Planchuelo-Gómez, Ana Echavarría Íñiguez, Ángel L. Guerrero-Peral

**Affiliations:** 1 Department of Medicine, Toxicology and Dermatology, Faculty of Medicine, University of Valladolid, 47002 Valladolid, Spain; gueneurol@gmail.com; 2 Headache Unit, Department of Neurology, Hospital Clínico Universitario de Valladolid, 47003 Valladolid, Spain; claudiagarciaruiz28@gmail.com (C.G.-R.); alvarosierramencia@gmail.com (Á.S.-M.); ygoinvestigacion@outlook.com (Y.G.-O.); andreareciogar99@gmail.com (A.R.-G.); anagonzalezcelestino@gmail.com (A.G.-C.); cris6gar@gmail.com (C.G.-I.); 3 Imaging Processing Laboratory, Escuela Superior de Telecomunicaciones (ETSI), Universidad de Valladolid, 47002 Valladolid, Spain; a.planchuelo.gomez@gmail.com; 4 Department of Neurology, Hospital Universitario de Burgos, 09006 Burgos, Spain; anaechavarria93@gmail.com

**Keywords:** headache disorders, COVID-19, drug therapy, amitriptyline, migraine, botulinum toxins, vaccine

## Abstract

To describe the need and effectiveness of acute and preventive medications in a series of 100 consecutive patients referred due to COVID-19-related headaches. Patients were aged 48.0 (standard deviation (SD): 12.4), 84% were female, and 56% had a prior history of headache. The most common headache phenotype was holocranial (63%), frontal (48%), pressing (75%), of moderate intensity (7 out of 10), and accompanied by photophobia (58%). Acute medication was required by 93%, with paracetamol (46%) being the most frequently used drug, followed by ibuprofen (44%). The drugs with the highest proportion of a 2 h pain-freedom response were dexketoprofen (58.8%), triptans (57.7%), and ibuprofen (54.3%). Preventive treatment was required by 75% of patients. The most frequently used drugs were amitriptyline (66%), anesthetic blockades (18%), and onabotulinumtoxinA (11%). The drugs with the highest 50% responder rate were amitriptyline (45.5%), mirtazapine (50%), and anesthetic blockades (38.9%). The highest 75% responder rate was experienced following onabotulinumtoxinA (18.2%). In conclusion, most patients required acute medication, with triptans and non-steroidal anti-inflammatory drugs achieving the best responses. Three-quarters of patients required preventive medication. The most frequently used drug was amitriptyline, which obtained the best results. In some treatment-resistant patients, anesthetic blockades and onabotulinumtoxinA were also beneficial.

## 1. Introduction

Headache is one of the most frequent symptoms of both acute coronavirus disease 2019 (COVID-19) and the post-COVID condition. It occurs in 23–47% of patients during the acute phase of the disease [1,2,3] and in 8–19% of patients beyond three months [1,4]. In the patients who experienced headaches, it was described as the most disabling symptom of the disease [2,5]. Distinctively from other symptoms that may persist and concur following the acute phase of the disease [6,7], headache is a treatable long COVID symptom [7,8].

Headache is an early symptom of COVID-19, which in most cases is present within the first 96 h of the disease [2,9]. Its clinical phenotype is typically holocranial, with frontal and temporal predominance, pressing quality, moderate-to-severe intensity, and worsening with physical activity. In a substantial proportion of cases, it is accompanied by photophobia, phonophobia, or nausea [2,5,10,11,12,13]. The headache phenotype fulfills the International Classification of Headache Disorders (ICHD) [14] criteria for tension-type headache in 54% of cases and for migraine in 25% [5].

Despite the rising burden of people suffering from COVID-19-related headaches, evidence regarding their acute and preventive treatment is almost inexistent [6,15,16,17]. We aimed to describe the effectiveness of the most frequently employed acute and preventive treatments in a real-world setting.

## 2. Materials and Methods

This is an observational descriptive study with a case series design. The study adhered to the Strengthening the Reporting for Observational Studies in Epidemiology (STROBE) guidance [18].

This study was conducted in the Headache Unit of Hospital Clínico Universitario, Valladolid, Spain, a third-level public hospital with a reference population of 261,000 inhabitants that serves as a headache reference center for a population of 2,700,000. Referrals can be made directly from primary care, from specialized care, or from general neurology consults. The waiting time between the referral and the evaluation ranged between 2 and 12 weeks during the study period. Patients were evaluated by three neurologists specializing in headache medicine.

### 2.1. Study Outcomes

The primary outcome was the 50% responder rate of the preventive treatments, defined as the proportion of patients who present a 50% reduction in the number of headache days per month, evaluated between weeks 8 and 12 after the onset of treatment, compared with the month prior to the treatment use. This outcome was adapted from the International Headache Society (IHS) guidelines for controlled trials of preventive treatment [19].

The secondary outcomes included the following: (1) to describe the proportion of patients that required acute or preventive treatment; (2) to describe the 30% and 75% responder rates of the preventive treatments, evaluated between weeks 8 and 12 [20]; and (3) to describe the response to the acute treatments, according to the IHS guidelines [19].

### 2.2. Eligibility Criteria

Patients were included if they (1) had a confirmed COVID-19 diagnosis, according to a real-time positive polymerase chain reaction test from a nasal or oropharyngeal swab sample and/or a positive result of IgM serum antibody testing in a patient with typical clinical symptoms; (2) presented headache during the course of the disease; (3) had been referred to the Headache Unit due to COVID-19-related headache. Cases were excluded if they (1) had any neurological or psychiatric condition resulting in cognitive impairment that could impede the description of the headache or the effectiveness of the treatments; (2) had language or speech disorders or insufficient performance of Spanish language; (3) died during the follow-up.

In patients with a prior history of headache, COVID-19-related headache was diagnosed, according to the ICHD-3, when there was a close temporal relationship between the COVID-19 infection and worsening of the headache, which had to present at least a two-fold or greater increase in frequency and/or severity in close temporal relationship with the COVID-19 infection [14].

### 2.3. Recruitment, Sampling, and Sample Size

The recruitment was based on a non-probabilistic approach. All consecutive patients referred to the Headache Unit were screened for eligibility. A sample of the first 100 patients was considered representative, with no formal sample size calculation due to the prior absence of evidence regarding acute or preventive treatment of COVID-19-related headaches. Patients were referred to the Headache Outpatient Clinic from primary care, other specialties, and other neurology consults. The need for acute and preventive treatment was based on the responsible physician’s opinion.

### 2.4. Study Period

Patients that had been infected between 1 March 2020, and 31 January 2022, were evaluated. To ensure a minimum follow-up of three months after the evaluation, patients were evaluated until 30 April 2022.

### 2.5. Intervention

In the first visit, an in-person evaluation by a headache expert was performed in all cases. A standardized questionnaire, based on previous studies [2,4], was administered. In the follow-up visits, either telephone or in-person visits were conducted, depending on the responsible physician’s judgment. Demographic variables included age and sex. The headache phenotype was described at the moment when the patients were evaluated. Clinical variables addressed family history of headache, prior history of headache, comorbidities (depression, anxiety, insomnia, or other chronic pain syndromes), and vaccination status against COVID-19. Concerning the COVID-19 infection, the time elapsed between the infection and the evaluation was assessed, as was the presence of fever, anosmia, or pneumonia as COVID-19 manifestations and the severity of COVID-19 (as defined in the Appendix A). The evaluation of the COVID-19-related headache included location (hemicranial, holocranial), topography (frontal, temporal, parietal, occipital, periocular, facial, cervical, or vertex), quality of pain (pressing, throbbing, stabbing, electric, burning), intensity of the headache in a numerical rating scale (0: no pain, 10: worst possible pain), associated symptoms (photophobia, phonophobia, osmophobia, nausea, vomiting, cranial autonomic symptoms), worsening of the headache by physical activity, and frequency of headache (number of headache days per month, number of acute medication days per month). Patients were allowed to report several locations of the headache and multiple locations of the pain. A headache diary was given to all patients to depict the frequency and intensity of headaches in order to evaluate the response to treatment. Treatments employed prior to the referral and those therapies prescribed in specialized care were evaluated. The follow-up duration of the included patients was also assessed. Since there were no guidelines or evidence-based treatments for COVID-19-related headaches, drugs were used off-label and based on the responsible physician’s preference and opinion. In some cases, the drugs were used based on the patients’ phenotype, and in other cases, the drugs were selected as empirical drug trials.

### 2.6. Acute Medication

The evaluation included the number of types of drugs employed and the clinical response. To evaluate the response to the acute medications, pain freedom at 2 h was employed [19]. Since patients reported the results following multiple uses of each drug, the response to each drug was classified according to patient and headache diary criteria, based on the proportion of uses where subjects became pain-free at 2 h, stratified into the following ranges: 0–30%, 31–50%, 51–75%, and >75% of uses.

### 2.7. Preventive Medication

The number and type of preventive medications were gathered. To describe the clinical response, the responder rate was estimated, defined as the percent reduction from baseline in the number of headache days per month between weeks 8 and 12, compared with the month prior to the use of the treatment. The 30%, 50%, and 75% responder rates were estimated per treatment. In the case of treatments that were used by more than 10 patients, the 50% and 75% responder rates were compared between patients with and without prior history of migraine and tension-type headache. Tolerability to treatments was also assessed, reporting treatment-emergent adverse events.

### 2.8. Ethics

This study was approved by the Valladolid East Ethics Review Board (PI 21-2499-TFG), and patients gave their consent prior to any study intervention. This study was conducted in accordance with the Declaration of Helsinki principles.

### 2.9. Statistical Analysis

Since the sample size was 100, qualitative and ordinal variables are described as percentages. When sub-groups or categories with missing data are reported, fractions are reported (with the denominator being the total number of cases) along with the percentage. Quantitative variables are reported as mean and standard deviation (SD) or median and inter-quartile range (IQR), depending on the type of distribution. Normality was assessed with the Kolmogorov–Smirnov test. In the hypothesis testing, a two-tailed Student *t*-test was used, setting a *p* value of 0.05 as the threshold for statistical significance when the distribution was normal and homoscedascidity was observed. The power of the study was estimated assuming a Type I error rate of 5%, with a true proportion of patients responding to amitriptyline reported as 44% in previous literature [4] and a 19% proportion of spontaneous resolution of COVID-19-related headache as the null hypothesis proportion [21]. With a sample size of 100 patients, the study power was estimated to be 0.99.

## 3. Results

Patients were aged 47.99 (SD: 12.42), and 84% were female. A family history of headaches was reported in 25% of cases. A prior history of headache was present only in 56% of patients, with migraine in 34%, tension-type headache in 12%, and other headache disorders in 15%. All patients with a prior history of migraine had a headache frequency within the range of episodic migraine at the time of COVID-19. Preventive drugs had been previously used by 11/34 (32.3%), being effective in 9/11 (81.8%) cases. The most frequently used drugs were amitriptyline in six patients; topiramate in five; betablockers in four patients; and gabapentin, flunarizine, and Zonisamide in one patient each. Patients had a prior history of anxiety (42%), insomnia (37%), depression (26%), and other painful disorders (26%). At the moment of the infection, COVID-19 vaccines had been administered to 8% of patients, including a single dose (4%), two doses (3%), and three doses (1%). Vaccinated patients had not been infected prior to their vaccination. The median follow-up duration was 6 months [IQR: 3–9 months; range: 3–21 months].

### 3.1. COVID-19 Infection

The time elapsed between the infection and the clinical evaluation was 7.16 (SD: 4.05) months. During the acute phase, 63% of patients had a fever, and 56% had anosmia. COVID-19 severity corresponded to mild illness (73%), pneumonia (21%), severe pneumonia (4%), and acute distress respiratory syndrome (2%). Two patients were admitted to the intensive care unit.

### 3.2. COVID-19-Related Headache

All patients fulfilled the criteria for *9.2.2.2 chronic headache attributed to systemic viral infection* (10). The most common headache phenotype was holocranial (63%), frontal (48%), and pressing (75%). The median intensity of the headache was 7 (IQR: 7–8.5). Table 1 summarizes the clinical phenotype of the headaches.

### 3.3. Acute Medication

**Figure 1:**
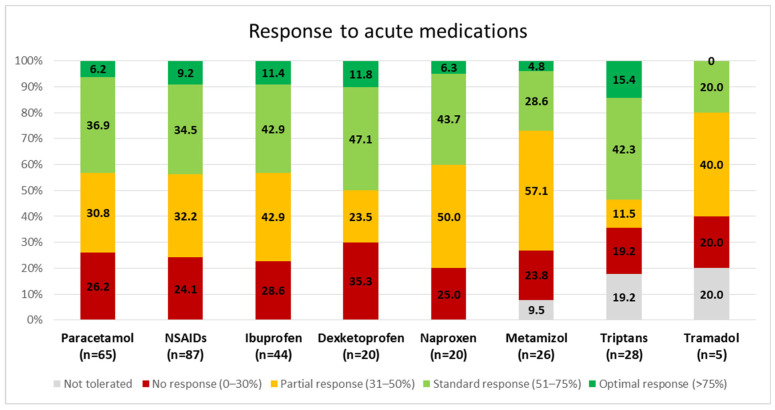
Response to the acute medications. Only treatments being used by at least four patients are represented. NSAIDs: non-steroidal anti-inflammatory drugs.

Ninety-three patients required at least one acute treatment. Patients required at least two acute treatments in 63% of cases, at least three in 33% of cases, at least four in 16% of cases, at least five in 9% of cases, at least six in 6% of cases, and at least seven in 1% of cases. The most frequently employed treatment was paracetamol (46%), followed by ibuprofen (44%), triptans (28%), metamizol (26%), naproxen and dexketoprofen (20% each), tramadol (5%), dihydroergotamine (2%), diclofenac, codeine, aspirin, and celecoxib (1% each). The drugs with the highest proportion of patients reporting consistently (>50% of times) achieving a pain-freedom response two hours after use were dexketoprofen (58.8%), triptans (57.7%), ibuprofen (54.3%), and paracetamol (43.1%). Figure 1 represents the proportion of patients reporting each type of response.

### 3.4. COVID-19 Infection

**Figure 2:**
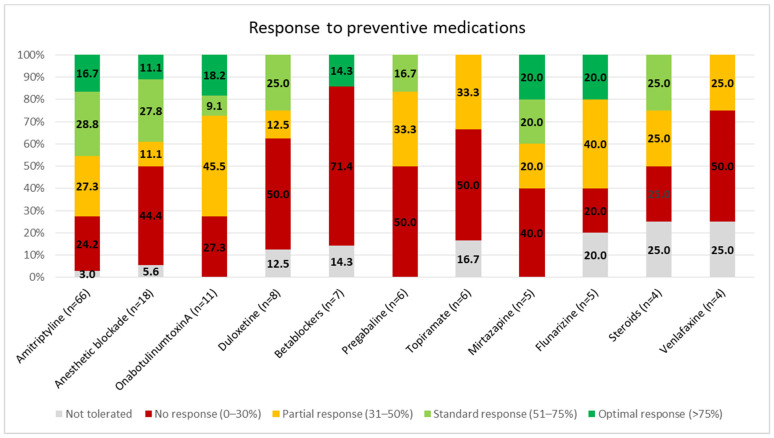
Response to the preventive medications. Only treatments being used by at least four patients are represented.

Preventive treatment was required in 75% of patients. Patients required at least two preventive treatments in 32% of cases, at least three in 17% of cases, at least four in 10% of cases, at least five in 5% of cases, at least six in 3% of cases, and at least seven in 1% of cases. The most frequently used drug was amitriptyline (66%), followed by anesthetic blockades (18%) and onabotulinumtoxinA (11%). The most frequently used drug as a first choice was amitriptyline in 53/75 (70.6%) cases, followed by anesthetic blockades in 9/75 (12%) cases. Figure 2 represents the proportion of patients who reported each type of response to the preventive medications. Table 2 represents the specific results per drug. Supplementary Figure S1 shows the specific drugs that were selected as first, second, and third choices in each case.

### 3.5. Responder Rate

**Figure 3:**
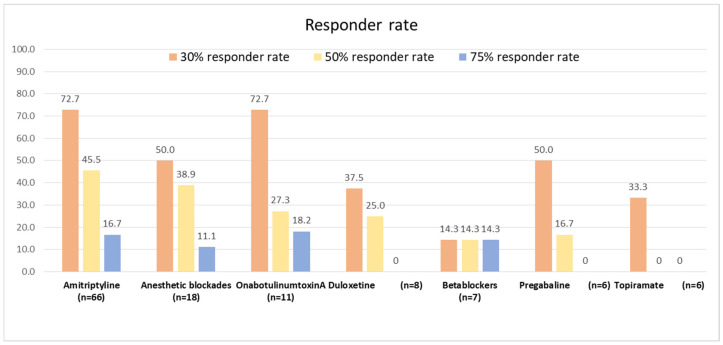
The 30%, 50%, and 75% responder rates for the most frequently employed treatments.

The drug with the highest 50% responder rate was amitriptyline (45.5%), followed by mirtazapine (50%) and anesthetic blockades (38.9%). The highest 75% responder rate was achieved by onabotulinumtoxinA (18.2%), and the highest 30% responder rate was observed following amitriptyline and onabotulinumtoxinA (72.7% in both cases). A 75% responder rate was more frequent in patients with a prior history of migraine than in those without a prior history of migraine (30.4% vs. 9.3%, *p* = 0.028). There were no further statistically significant differences between patients with a prior history of migraine or tension-type headache and those without a prior history of headache (Supplementary Figure S2). Figure 3 summarizes the responder rates for the most frequently employed drugs.

### 3.6. Tolerability

Adverse events were observed in 23/66 (34.8%) patients treated with amitriptyline, including somnolence in 14/66 (21.2%), gastrointestinal pain in 6/66 (9.1%), weight gain, lightheadedness, or dry mouth in 3/66 (4.6%) each, and nausea in 1/66 (1.5%). Patients treated with anesthetic blockades reported local pain in 4/18 (22.2%) cases, and patients treated with onabotulinumtoxinA reported neck pain, headache, and aesthetic effects in 2/11 (18.2%) cases each.

## 4. Discussion

In the present study, we assessed the need and response to acute and preventive treatment in patients with COVID-19-related headaches. The first 100 COVID-19 survivors that were referred to our Headache Unit due to COVID-19-related headaches were studied and characterized. The main findings were that 93% of patients required acute medication, with paracetamol being the most frequently used treatment and triptans being the symptomatic treatment with the best response rate. Three-quarters of patients required preventive medication, with amitriptyline being both the most frequently used drug and the treatment with the best responder rates.

In this study, the clinical phenotype of the headaches was described and the moment of the evaluation, and it partially differed from that of the patients who reported headaches during the acute phase of the disease [2,4,7,8,9,10,11,12,13], perhaps related to a possible selection of patients that required treatment. In our setting, more than 458 patients with headaches were evaluated, and less than 25% of them were referred to our Headache Unit [2]. In this sample, headache was more frequently reported as hemicranial, throbbing, or stabbing and had a higher frequency of typical migraine symptoms. The reasons why headaches persist in some COVID-19 survivors are unclear. In a study that assessed the prospective duration of headache in 905 COVID-19 patients who presented headaches during the acute phase of the disease, patients with persistent headache at 9 months had a higher frequency of throbbing pain, photophobia or phonophobia, and worsening by physical activity [3], which could suggest some degree of predisposing migraine biology. In our sample, 56% of patients had a prior history of headache, numbers in line with the global estimated prevalence of active headache disorders [22], but 34% had a prior history of migraine, numbers that are two-fold higher than the highest prevalence rate in our country [23] and higher than any estimated rate worldwide [22]. However, the research question of this study was not whether patients with a prior history of headache or migraine are more prone to develop a persistent headache after COVID-19. The higher observed proportion of patients with a prior history of migraine could be caused by a selection bias, since these patients were referred to a headache unit. The response to treatment may support this hypothesis, since paracetamol was not effective in most cases, while triptans and non-steroidal anti-inflammatory drugs (NSAIDs) were the most effective treatments.

Despite the initial concern about the use of NSAIDs in patients with COVID-19 [24], further evidence confirmed their safety [25]. A prior study including 330 patients managed in an outpatient setting and 107 hospitalized patients reported the need for acute medication in 94% of patients with headaches during the acute phase of the disease [2], with paracetamol being the most frequently employed drug (92%), followed by ibuprofen (17%) and metamizole (12%). In that study, 19% of patients were acute treatment-resistant [2], numbers that are in line with another study that evaluated 97 patients that visited the emergency department due to COVID-19 and presented with headaches, with patients with mild-to-moderate headaches being better responders to acute treatment than patients with severe headaches (66% vs. 37%) [10]. In our study, triptans were the drug with the best response rate and may be considered, especially when the headache exhibits migraine-like features.

Regarding preventive treatment, this was needed by 75% of the patients that were evaluated. One of the main limitations of this study is the absence of a control arm. However, our results may be beneficial to select which treatments may be better suited for being studied in a double-blind randomized placebo-controlled trial (RCT). Nevertheless, prior evidence suggests that when a COVID-19-related headache has not resolved two months after the acute phase, it becomes persistent and adopts a chronic pattern [4]. Therefore, we evaluated the effectiveness of each treatment with the patients’ situation prior to the use of the therapy, in accordance with the IHS guidelines [10]. The selection of the preventive drugs may be influenced by the clinical phenotype of the headache, which presents similarities with tension-type headaches and migraines [2,5].

Amitriptyline is the first-line treatment for tension-type headaches [26] and one of the most frequently used oral preventive drugs for migraines [27]. In addition, it may be helpful in the treatment of insomnia [28], musculoskeletal [29] or neuropathic pain [30], or mood disorders [31]. To date, evidence of the effectiveness of amitriptyline in COVID-19-related headaches comes from a series of 3 patients [15] and a series of 48 patients [21], where the proportion of patients that presented a 50% responder rate was 44%, with a 30% responder rate of 50% and a 75% responder rate of 21%. A prior history of tension-type headaches and nausea was associated with a higher probability of response [21]. The results that have been reported in the literature and the findings of the present study are in line with the benefits that have been reported in RCTs for migraine. In these RCTs, the 50% responder rate in patients with migraine treated with amitriptyline was reported as 39% out of 59 patients [32] and 46% out of 95 patients [33], making amitriptyline the oral preventive drug that showed the best results in the network meta-analyses of RCTs evaluating oral preventive drugs for migraine [34].

Prior to the present study, evidence regarding other treatments was limited to a series of cases regarding steroids (n = 3) [16,35] or onabotulinumtoxinA (n = 2) [12]. Greater occipital nerve anesthetic blockades were used in the treatment of headaches associated with acute headaches in a series of 27 COVID-19 patients, with a decrease in the mean intensity of the headaches 10 days after the infiltration [36].

Our study leaves some unanswered questions, including the effectiveness of other novel therapies, such as anti-calcitonin gene-related peptide antibodies and antagonists, and the reasons for the treatment resistance that some patients present. If the pathophysiology of the post-COVID-19 condition is more related to the persistent activation of the immune system [37], the treatment approach should consider the use of immune modulators. On the other hand, the effects of rehabilitation [6] and psychological treatment [38] have not been addressed in our study. The assessment and treatment of COVID-19 patients must be multidisciplinary [39] and may address the management of other possible comorbidities, which in the case of neurological manifestations are among the most prevalent [40,41]. The long-term association between anosmia/ageusia and headache was not evaluated either. In addition, it remains unclear whether post-COVID-19 headache is a subtype of a new daily persistent headache or a chronic form of a disorder that in most patients is restricted to the acute phase [42,43].

The present study has relevant limitations. The study findings may not be generalizable to other settings due to their single-center nature. We could not estimate the proportion of patients that had been infected during the study period, and there may be a selection bias, with patients being probably selected towards a more severe and treatment-resistant pattern. The sample size was limited, and the heterogeneity of the patients did not allow for an evaluation of the effectiveness of some treatments and the patients’ characteristics. There was no control arm; therefore, some patients could have improved by chance. The specific COVID-19 variant was not assessed, and most patients had not been vaccinated, which may impact the COVID-19-related headache duration and may vary the treatment response. This was related to the study period, which recruited most patients prior to the vaccination campaigns. Future RCTs should evaluate the short- and long-term efficacy of the acute and preventive medications for COVID-19-related headaches, which may consider the results of the present study to estimate the sample size and select the most suitable therapies. In our study, only the main endpoints related to acute and preventive medication were evaluated. Future studies may also evaluate additional endpoints, such as sustained pain freedom, the absence of the most bothersome symptom, or headache intensity [15].

## 5. Conclusions

In the present series of COVID-19 survivors who were evaluated in our Headache Unit around seven months after the acute phase, acute medication had been employed by most patients, with triptans and NSAIDs being the therapies with the best results. Nevertheless, more than 40% of patients were acute treatment-resistant. Preventive treatment was needed by three-quarters of patients, with amitriptyline being the most frequently employed therapy and the drug with the best responder rate.

## Figures and Tables

**Table 1:** Variables related to the clinical phenotype of the headaches.

Variable	Proportion (n = 100)
**Headache location**	
Strictly holocranial	63%
Strictly hemicranial	15%
Both holocranial and hemicranial	22%
**Headache topography**	
Frontal	48%
Occipital	36%
Temporal	27%
Periocular	21%
Parietal	15%
Vertex	9%
Cervical	5%
Facial	4%
**Quality of pain**	
Pressing	75%
Throbbing	27%
Stabbing	27%
**Associated symptoms**	
Photophobia	58%
Phonophobia	47%
Osmophobia	10%
Cranial autonomic symptoms	7%
Nausea	36%
Vomiting	7%
Worsening by physical activity	46%

**Table 2:** Specific response to each preventive drug.

Drug n, %	Not Tolerated	No Response (0–30%)	Partial Response (31–50%)	Standard Response (50–75%)	Optimal Response (>75%)
**Amitriptyline (n = 66)**	2 (3.0%)	16 (24.2%)	18 (27.3%)	19 (28.8%)	11 (16.7%)
**Anesthetic blockade (n = 18)**	1 (5.6%)	8 (44.4%)	2 (11.1%)	5 (27.8%)	2 (11.1%)
**OnabotulinumtoxinA (n = 11)**	0 (0%)	3 (27.3%)	5 (45.4%)	1 (9.1%)	2 (18.2%)
**Duloxetine (n = 8)**	1 (12.5%)	4 (50%)	1 (12.5%)	2 (25%)	0 (0%)
**Betablockers (n = 7)**	1 (14.3%)	5 (71.4%)	0 (0%)	0 (05)	1 (14.3%)
**Pregabaline (n = 6)**	0 (0%)	3 (50%)	2 (33.3%)	1 (16.7%)	0 (0%)
**Topiramate (n = 6)**	1 (16.7%)	3 (50%)	2 (33.3%)	0 (0%)	0 (0%)
**Mirtazapine (n = 5)**	0 (0%)	2 (40%)	1 (20%)	1 (20%)	1 (20%)
**Flunarizine (n = 5)**	1 (20%)	1 (20%)	2 (40%)	0 (0%)	1 (20%)
**Steroids (n = 4)**	1 (25%)	1 (25%)	1 (25%)	1 (25%)	0 (0%)
**Venlafaxine (n = 4)**	1 (25%)	2 (50%)	1 (25%)	0 (0%)	0 (0%)
**Gabapentine (n = 1)**	0 (0%)	0 (0%)	0 (0%)	1 (100%)	0 (0%)
**Zonisamide (n = 1)**	0 (0%)	0 (0%)	1 (100%)	0 (0%)	0 (0%)
**Candesartan (n = 1)**	0 (0%)	0 (0%)	1 (100%)	0 (0%)	0 (0%)

## Data Availability

The anonymized datasets supporting the conclusions of this study are available for other researchers upon request to the corresponding author.

## Supplementary Materials

The following supporting information can be downloaded at https://www.mdpi.com/article/10.3390/life14070910/s1, Supplementary Figure S1: Order of use of preventive medications. Supplementary Figure S2: Response rate depending on the prior history of migraine or tension-type headache.

## Appendix A

**Table A1:** Severity of COVID-19 disease according to the American Thoracic Society guidelines for community-acquired pneumonia ^1^.

Severity	Description
Mild illness	Patients with uncomplicated upper respiratory tract viral infection symptoms and non-specific symptoms such as fever, fatigue, cough (with or without sputum production), anorexia, malaise, muscle pain, sore throat, dyspnea, nasal congestion, diarrhea, nausea, or vomiting.
Pneumonia	Presence of pneumonia but no signs of severe pneumonia and no need for supplemental oxygen. CURB scale ≤ 1.
Severe pneumonia	Confirmed respiratory infection, plus one of the following: Respiratory rate > 30 breaths/min. Severe respiratory distress. SpO_2_ ≤ 93% on room air.
Acute respiratory distress syndrome (ARDS)2	**Onset:** within 1 week of a known clinical insult or new or worsening respiratory symptoms. **Chest imaging** (radiograph, CT scan, or lung ultrasound): bilateral opacities, not fully explained by volume overload, lobar or lung collapse, or nodules. **Origin of pulmonary infiltrates**: respiratory failure not fully explained by cardiac failure or fluid overload. Need objective assessment (e.g., echocardiography) to exclude hydrostatic causes of infiltrates/edema if no risk factor present. **Oxygenation impairment in adults**: Mild ARDS: 200 mmHg < PaO_2_/FiO_2_a ≤ 300 mmHg (with PEEP or CPAP ≥ 5 cmH_2_O, or non-ventilated) Moderate ARDS: 100 mmHg < PaO_2_/FiO_2_ ≤ 200 mmHg (with PEEP ≥ 5 cmH_2_O, or non-ventilated) Severe ARDS: PaO_2_/FiO_2_ ≤ 100 mmHg (with PEEP ≥ 5 cmH_2_O, or non-ventilated) When PaO_2_ is not available, SpO_2_/FiO_2_ ≤ 315 implies ARDS (including in non-ventilated patients).

Sp: saturation percentage. ARDS: acute respiratory distress syndrome. CT: Cranial Tomography. PaO_2_: partial pressure of oxygen. FiO_2_: fraction of inspired oxygen. PEEP: positive end-expiratory pressure. CPAP: continuous positive airway pressure. ^1^ Source: [44,45].
