# Supplementary material for: Acute and Preventive Treatment of COVID-19-Related Headache: A Series of 100 Patients
Source: Life (Basel). 2024 Jul 22;14(7):910. doi: 10.3390/life14070910 (PMC11277981; doi:10.3390/life14070910)

Supplementary Materials:

Supplementary Figure S1: Order of use of preventive medications in the entire study sample.

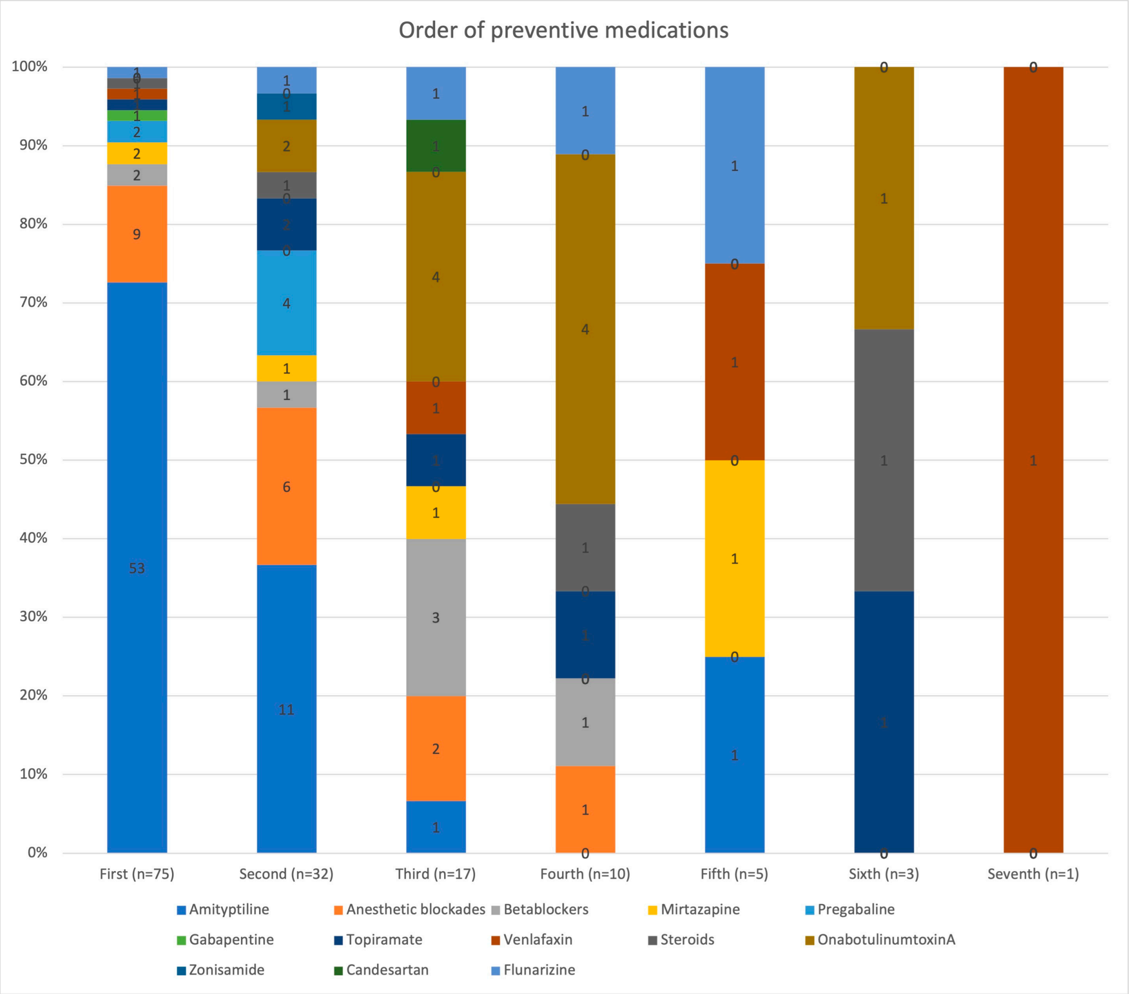

Supplementary Figure S2: Response rate in patients depending on prior history of headache.

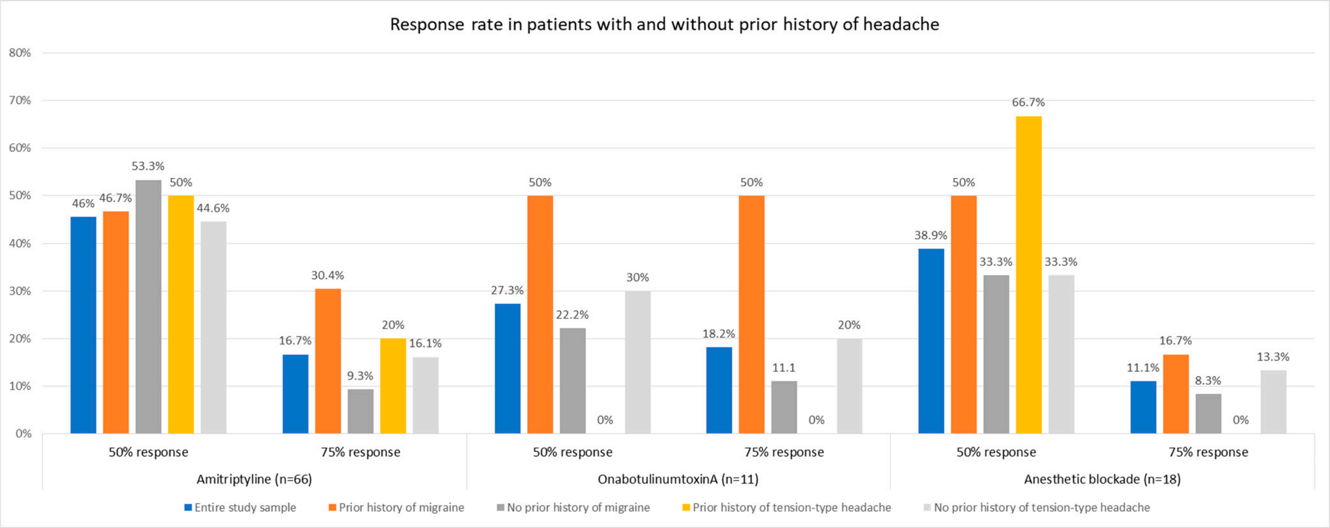

Supplement: Supplementary file 1 [file life-14-00910-s001.zip › life-3069269-supplementary.pdf]
